# Co-culture shock

**DOI:** 10.1038/s41420-025-02934-7

**Published:** 2026-01-21

**Authors:** James W. Behan, Steven D. Mittelman

**Affiliations:** 1 Department of Dermatology, The Permanente Medical Group, Pleasanton, CA USA; 2 Division of Pediatric Endocrinology, UCLA Children’s Discovery and Innovation Institute, David Geffen School of Medicine, UCLA, Los Angeles, CA USA

**Keywords:** Acute lymphocytic leukaemia, Cancer models

Dear Editor,

We read with great interest the recent article published by Zinngrebe et al. [1], entitled “Bridging the marrow: a co-culture-platform of leukemia cells and MS5-derived stromal cells or adipocytes.” We were pleased to see the authors interrogating the relationships between adipocytes and acute lymphoblastic leukemia (ALL) cells, as we have been studying this relationship since we first reported it in 2009 [2], and there is still much to learn.

The authors developed a model of MS5-derived adipocytes co-cultured with human acute lymphoblastic leukemia (ALL) cell lines or primary ALL cell samples. With this specific model, they found that adipocytes make ALL cells more sensitive to the chemotherapies daunorubicin and dexamethasone. These results appear to contradict findings by ours [2–6] and other groups [7–11] showing that adipocytes *protect* ALL cells from chemotherapies. The authors suggest that differences may be secondary to confounding effects of substances contained in the adipogenic media used to differentiate cells into adipocytes and infer that perhaps other groups did not use “well-controlled culture conditions,” such as those they established in their study.

We agree with the authors that differences in our experimental protocols likely explain why their results contrast with ours and others. No single model accurately reflects the in vivo ALL microenvironment, and all models have their strengths and weaknesses. This is why the use of multiple models is so important to support the rigor of one’s results. Indeed, we have described experiments using three different adipocyte cell lines (3T3-L1, OP9, or ChubS7) cultured with seven human (SD1, RS4;11, RCH, Nalm6, Molt4, SupB15, and BV173) and one murine (8093) ALL cell line, with treatment with nine different chemotherapies (vincristine, dexamethasone, L-asparaginase, PEG-asparaginase, erwinase, daunorubicin, doxorubicin, nilotinib, and mitoxantrone) [2–6]. These studies were done with direct co-culture, indirect coculture with TransWells, and in some cases with media conditioned by adipocytes alone or with ALL cells. Other groups have also shown protection of ALL cells from chemotherapy by 3T3-L1 [8], MS5 [7], OP-9 [11] and human MSC-derived [9, 10] adipocytes. Importantly, we confirmed many of our findings using murine [12] and human [5] adipose tissue explants ex vivo. Further, our in vitro results are consistent with the observation that obesity confers a worse ALL treatment outcome in both pre-clinical models [2, 4, 13] and clinical studies [14–16].

Zinngrebe et al. used an adipocyte model derived from the MS5 cell line and cocultured with ALL cell lines for 96 hours or patient-derived xenograft (PDX) ALL cells for 7 days. Adipocytes were transferred from adipogenic medium (DMEM/F12 with adipogenic additives and 10% FBS) into serum free DMEM/F12 in the coculture conditions. ALL cells were transferred from αMEM with 10% FBS into this serum free DMEM/F12, and so experienced withdrawal of FBS along with decreased concentrations of nutrients, including glucose (55.5→7.5 mM) and amino acids (34→7 mM). These changes likely had dramatic but difficult-to-quantify effects on both cell types. Further, chemotherapy sensitivity experiments lasted 4-7 days in this serum-free media. It does not appear that media was changed or refreshed, which if not, could lead to decreased fuel availability, buildup of metabolic waste products, and pH changes. Thus, what started as chemically-defined media may have ended with dramatically different compositions in the different experimental coculture conditions over the course of these experiments.

We and others who have tested chemotherapy sensitivity in ALL-adipocyte coculture have used 24-72 hour experiments [2–11], and all but one [10] used media with serum. While serum is not chemically-defined and can vary significantly by source and batch, its growth factors and other components better mimic the in vivo microenvironment of ALL cells, and so assessments of chemotherapy sensitivity may better reflect what is happening in patients.

Finally, the authors stated that studies on adipocytes and ALL “often lack the information as to whether essential washing steps were performed before co-culture.” In our initial study reporting on the relationship between ALL and adipocytes [2] we described that dexamethasone and isobutylmethylxanthine were removed from the adipogenic media on day +2, adipocytes were used for coculture between days +7 and +14, and media changes were performed every 2 d until use. Thus adipocytes underwent at least two media changes and five days in culture after the steroid was removed. Besides, if some dexamethasone had still been present during the coculture, its confounding effect would have been to induce more cytotoxicity to chemotherapy, not less as we reported.

Thus, we believe that the Zinngrebe et al. study adds an interesting, contrasting result to the body of literature examining the relationships between adipocytes and ALL. However, since their culture conditions were so different than ours and the published literature, we do not believe this casts doubt on the findings that adipocytes protect ALL cells from chemotherapy first reported by us and since confirmed by four other groups. We hope the authors will continue to pursue their interesting findings.

Signed,

James W. Behan, MD, and Steven D. Mittelman, MD, PhD
